# Appropriateness of Antibiotic Prescribing in US Emergency Department Visits, 2016–2021

**DOI:** 10.1017/ash.2024.79

**Published:** 2024-05-14

**Authors:** Joseph Benigno Ladines-Lim, Michael A. Fischer, Jeffrey A. Linder, Kao-Ping Chua

**Affiliations:** 1 Departments of Internal Medicine and Pediatrics, University of Michigan, Michigan Medicine, Ann Arbor, MI, USA; 2 Section of General Internal Medicine, Boston Medical Center, Boston University Chobanian & Avedisian School of Medicine, Boston, MA, USA; 3 Division of General Internal Medicine, Department of Medicine, Northwestern University Feinberg School of Medicine, Chicago, IL, USA; 4 Susan B. Meister Child Health and Evaluation Research Center, Department of Pediatrics, University of Michigan Medical School, Ann Arbor, MI, USA

## Abstract

In this national analysis of US emergency department visits with antibiotic prescribing during 2016–2021, 27.6% of visits resulted in inappropriate antibiotic prescribing: 14.9% had diagnosis codes plausibly antibiotic-related (eg, acute bronchitis), suggesting actual inappropriate prescribing, and 12.6% had diagnosis codes not plausibly antibiotic-related (eg, hypertension), suggesting poor coding quality.

## Introduction

Inappropriate outpatient antibiotic prescribing contributes to antimicrobial resistance.^1^ Antibiotics are commonly prescribed during emergency department (ED) visits.^2–4^ Although a prior analysis found that 23%–30% of ED and office visit antibiotic prescriptions during 2010–2015 were inappropriate,^5^ there are no recent national data on antibiotic prescribing appropriateness in EDs specifically.

Inappropriate ED antibiotic prescriptions could be associated with diagnosis codes for infectious conditions that are plausible yet inappropriate indications for antibiotics (eg, acute bronchitis). Alternatively, they could be associated only with diagnosis codes for conditions not plausible for antibiotics (eg, hypertension), as one study found in 18% of office visit antibiotic prescriptions in 2015.^6^ To our knowledge, no comparable studies in EDs have been performed. Conducting such studies could facilitate understanding of how coding may affect estimates of inappropriate antibiotic prescribing rates in EDs.

Using national ED visit data from 2016–2021, we estimated the proportion of visits with inappropriate antibiotic prescribing. Additionally, we estimated the proportion of visits with inappropriate antibiotic prescriptions that did and did not have plausible antibiotic indications.

## Methods

We analyzed the National Hospital Ambulatory Medical Care Survey (NHAMCS), a nationally representative survey of ED visits fielded by the Centers for Disease Control and Prevention.^5,7^ Data include patient characteristics, up to five diagnosis codes abstracted by chart review, and information on up to 30 medications prescribed or administered. Details on the NHAMCS are published elsewhere.^8^ Because data are de-identified, the Institutional Review Board of the University of Michigan Medical School exempted this study from human patients review.

**Figure 1.**
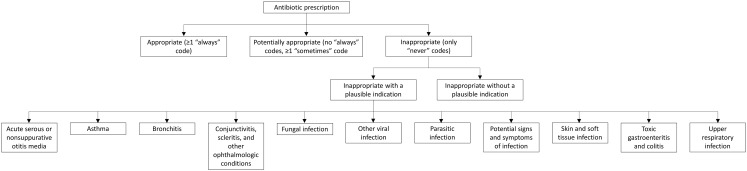
Categorization and subcategorization of appropriateness of antibiotic prescriptions. All categories and subcategories are mutually exclusive.

We included ED visits with ≥1 oral antibiotic prescription, excluding visits only associated with non-billable diagnosis codes and visits resulting in transfer to another facility (see Supplemental Methods 1 for details). Following our prior study, we classified each diagnosis code for the visit as “always,” “sometimes,” or “never” justifying antibiotic use, using this to classify prescribing as appropriate, potentially appropriate, or inappropriate (Figure 1).^9^

Among visits with inappropriate prescribing in our sample, we found 2,698 unique “never” codes. We determined via consensus whether these could be plausible reasons for prescribing antibiotics (see Supplemental Methods 2), including non-bacterial infections and infections for which clinicians often inappropriately prescribe antibiotics. We assigned codes that could be plausible antibiotic indications to one of the following categories: acute serous or nonsuppurative otitis media; asthma; bronchitis; conjunctivitis, scleritis, and other ophthalmologic conditions; fungal infection; parasitic infection; potential signs and symptoms of infection; skin and soft tissue infection (eg, viral warts rather than cellulitis); toxic gastroenteritis and colitis (the only intra-abdominal infectious code in the sample); upper respiratory infection (URI); and other viral infection (Figure 1). We created the “potential signs and symptoms of infection” category to account for infection-related diagnosis codes that are not antibiotic indications by themselves (eg, fever, abdominal pain). If multiple “never” codes could be plausible antibiotic indications, we selected the first one listed. In a sensitivity analysis, we randomly selected the plausible “never” code rather than the first one.

We conducted analyses for the overall sample and then separately among children (0–17 yr), adults (18–64 yr), and older adults (≥65 yr). We accounted for the complex design of NHAMCS by using survey weights and design-based variance estimators. We used R version 4.3.0 (R Foundation for Statistical Computing, Vienna, Austria).

## Results

There were 819,395,799 weighted ED visits (based on 105,212 unweighted sampled visits). We included 152,449,442 visits (18.6%) with ≥1 antibiotic prescription, with 20.8% for children, 61.7% for adults, and 17.5% for older adults. Supplemental Table 1 reports additional characteristics.

Among visits with antibiotic prescriptions, 27.6% had inappropriate prescribing, with 14.9% and 12.7% with and without a plausible antibiotic indication, respectively. Thus, 54.0% of visits with inappropriate antibiotic prescribing had a plausible antibiotic indication, while 46.0% did not (Supplemental Figure 1).

Among visits with inappropriate antibiotic prescribing and a plausible indication, the most frequent indications were potential signs and symptom of infection (54.0%), bronchitis (17.4%), and URI (10.4%) (Supplemental Table 2). The most frequent potential signs and symptoms of infection were abdominal pain, headache, nausea with vomiting, dyspnea, and fever (Supplemental Table 3). Among visits with inappropriate antibiotic prescribing and no plausible indication, the most frequent diagnosis codes were essential hypertension, chest pain, and joint pain (Supplemental Table 4).

**Figure 2.**
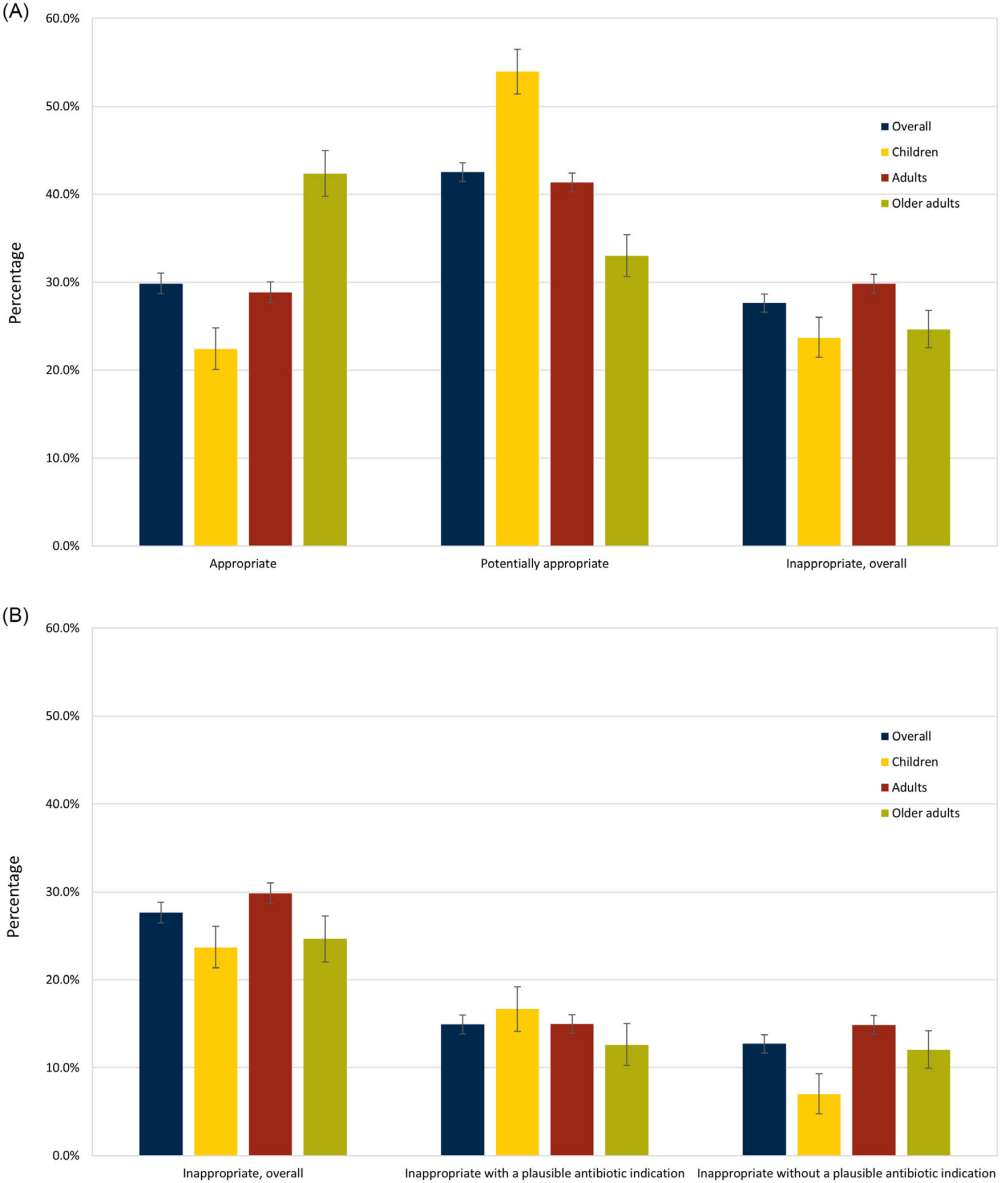
(A) Appropriateness of antibiotic prescribing in United States emergency departments, 2016–2021. (B) The proportion of visits with inappropriate antibiotic prescribing in United States emergency departments, 2016–2021, overall and with or without plausible antibiotic indications. First set of bars equals the sum of the proportions of the second and third sets of bars.

Figure 2 shows results by age group. Visits for adults had the highest prevalence of inappropriate antibiotic prescribing (29.8%, 95% CI: 28.8%–30.9%) versus children (23.7%, 95% CI: 21.4%–26.0%) and older adults (24.6%, 95% CI: 22.5%–26.8%). Inappropriate prescribing with a plausible indication was highest for children (16.7%, 95% CI: 14.6%–18.9%), followed by adults (15.0%, 95% CI: 14.0%–16.0%) and older adults (12.6%, 95% CI: 11.1%–14.2%). In contrast, inappropriate prescribing without a plausible antibiotic indication was lowest in children (7.0%, 95% CI: 5.9%–8.2%) versus adults (14.9%, 95% CI: 13.9%–15.9%) and older adults (12.0%, 95% CI: 10.6%–13.6%). The most frequent plausible antibiotic-inappropriate indications were potential signs and symptoms of infection, URI, and bronchitis in all age groups. Sensitivity analysis results were similar (Supplemental Table 5).

## Discussion

In this national analysis of ED visits with antibiotic prescribing during 2016–2021, 27.6% had inappropriate prescribing. Approximately 54.0% of these visits had plausible coded indications for antibiotics, while 46.0% did not.^5^ Findings suggest that ED antibiotic stewardship initiatives should focus both on reducing antibiotic prescribing for infectious, antibiotic-inappropriate conditions and on improving coding quality for antibiotic prescriptions.

We are unaware of any other more recent studies of inappropriate antibiotic prescribing in EDs using national data. The last most comparable study to our knowledge found that 23%–30% of ED and office visit antibiotic prescriptions during 2010–2015 were inappropriate.^5^ However, that study only focused on antibiotic prescribing during visits in which antibiotic-inappropriate infectious conditions (eg, acute bronchitis) were coded.

Among visits with inappropriate antibiotic prescribing and a plausible indication, the most frequent indication was potential signs or symptoms of infection (eg, fever and dyspnea), warranting future research. If these visits represent instances in which clinicians were unsure of the diagnosis but prescribed antibiotics regardless, stewardship initiatives might focus on safely minimizing unnecessary antibiotic prescribing with diagnostic uncertainty, such as using testing or scoring tools to determine the likelihood of actual bacterial infection.^2^ If these visits represent instances in which clinicians had diagnostic certainty but coded nonspecific signs or symptoms, initiatives should focus on improving coding quality.

Among ED visits with inappropriate antibiotic prescribing, almost half lacked plausible diagnosis codes. These may represent instances in which clinicians prescribed antibiotics appropriately but neglected to code the condition, or alternatively, prescribed antibiotics for antibiotic-inappropriate conditions and deliberately avoided coding these to avert scrutiny. Motivation for the latter behavior may be increased if antibiotic prescribing is assessed by quality measures reliant on diagnosis codes for case identification; considering how performance measures could have such unintended consequences also warrants future research.

This study had limitations. First, NHAMCS reports up to five ICD-10-CM diagnosis codes per visit. It is possible that antibiotic indications coded during the visit did not appear in the data. Second, concerns have been raised about the accuracy of chart abstraction in NHAMCS, although these concerns focused on under-capture of testing as opposed to mis-capture of diagnosis codes.^10^ Third, examining changes in inappropriate antibiotic prescribing during the COVID-19 pandemic was beyond our scope.

## Supporting information

Ladines-Lim et al. supplementary material 1 Ladines-Lim et al. supplementary material

Ladines-Lim et al. supplementary material 2 Ladines-Lim et al. supplementary material

## Supplementary material

The supplementary material for this article can be found at https://doi.org/10.1017/ash.2024.79.
