# Supplementary material for: Appropriateness of Antibiotic Prescribing in US Emergency Department Visits, 2016–2021
Source: Antimicrob Steward Healthc Epidemiol. 2024 May 14;4(1):e79. doi: 10.1017/ash.2024.79 (PMC11094377; doi:10.1017/ash.2024.79)

**Supplement Table of Contents**

**Supplemental Methods 1.** Methods for identifying antibiotics.

**Supplemental Methods 2.** Subclassifications of “never” diagnosis codes.

**Supplemental Table 1.** Demographic characteristics of sample by age group.

**Supplemental Table 2.** Most frequent indications among visits with inappropriate antibiotic prescribing and a plausible antibiotic indication.

**Supplemental Table 3.** Most frequent diagnosis codes among visits with inappropriate antibiotic prescribing in which the indication was a potential signs and symptoms of infection.

**Supplemental Table 4.** Most frequent diagnosis codes among visits with inappropriate antibiotic prescribing and no plausible antibiotic indication.

**Supplemental Table 5.** Most frequent indications among visits with inappropriate antibiotic prescribing and a plausible antibiotic indication, main versus sensitivity analysis.

**Supplemental Methods 1.** Methods for identifying antibiotics.

The National Hospital Ambulatory Medical Care Survey (NHAMCS) uses the Cerner Multum Lexicon Plus database to convert information on drugs described to therapeutic drug classes and agents. To identify eligible antibiotics in our study, we used Multum codes as shown below.

| **Antibiotic agents** | **Multum code** |
| --- | --- |
| Metronidazole | d00108 |
| Tinidazole | d04935 |
| Atovaquone | d01120 |
| Rifampin | d00047 |
| Cefaclor | d00081 |
| Cefadroxil | d00080 |
| Cefdinir | d04256 |
| Cefixime | d00072 |
| Cefpodoxime | d00095 |
| Cefprozil | d00073 |
| Cefuroxime | d00056 |
| Cephalexin | d00096 |
| Vancomycin | d00125 |
| Clindamycin | d00043 |
| Azithromycin | d00091 |
| Clarithromycin | d00097 |
| Erythromycin | d00046 |
| Rifaximin | d05294 |
| Linezolid | d04534 |
| Amoxicillin | d00088 |
| Amoxicillin-clavulanate | d00089 |
| Ampicillin | d00003 |
| Dicloxacillin | d00153 |
| Penicillin | d00116 |
| Ciprofloxacin | d00011 |
| Fluoroquinolones, miscellaneous | n08223 |
| Gatifloxacin | d04504 |
| Levofloxacin | d04109 |
| Moxifloxacin | d04500 |
| Ofloxacin | d00114 |
| Sulfamethoxazole | d00119 |
| Sulfamethoxazole-trimethoprim | d00124 |
| Sulfonamides, miscellaneous | c00015 |
| Doxycycline | d00037 |
| Minocycline | d00110 |
| Sarecycline | d09003 |
| Tetracycline | d00041 |
| Demeclocycline | d01068 |
| Oxytetracycline | d01070 |
| Fosfomycin | d04106 |
| Methenamine | d00106 |
| Nitrofurantoin | d00112 |
| Trimethoprim | d00123 |

**Supplemental Methods 2.** Subclassifications of “never” diagnosis codes.

Among all visits with inappropriate antibiotic prescriptions (i.e., only associated with “never” codes) in the sample, there were 2,698 unique “never” diagnosis codes. We further subclassified these codes as plausible indications for antibiotics or not (“Never, plausible” versus “Never, not plausible”). For those deemed plausible, we also include diagnosis subcategories, such as skin and soft tissue infection, potential signs and symptoms of infection, and upper respiratory infection, among others. We included these subclassifications in an attachment (see file entitled “ICD-10-CM_NHMACS_never_2016_2017_2018_2019_2020_2021.xlsx”). This file also contains the classification decisions used for the sensitivity analysis, in addition to those used for the main analysis.

**Supplemental Table 1.** Demographic characteristics of sample by age group. Subcategories may not sum up to total sample size due to rounding errors after use of survey weights and design-based variance estimators to account for the complex survey design.

| **Characteristic** | **Overall, No. (%)** (n = 152,449,442) | **Children, No. (%)**  (n = 31,714,726) | **Adults, No. (%)**  (n = 94,016,603) | **Older adults, No. (%)**  (n = 26,718,112) |
| --- | --- | --- | --- | --- |
| **Female sex** | 87,737,545 (57.6%) | 15,702,068 (49.5%) | 56,638,335 (60.2%) | 15,397,141 (57.6%) |
| **Age group, years** |  | | | |
| 0-1 | ··· | 6,631,523 (20.9%) | ··· | ··· |
| 2-5 | ··· | 9,857,892 (31.1%) | ··· | ··· |
| 6-11 | ··· | 8,583,401 (27.1%) | ··· | ··· |
| 12-17 | ··· | 6,641,908 (20.9%) | ··· | ··· |
| 18-25 | ··· | ··· | 20,222,873 (21.5%) | ··· |
| 26-34 | ··· | ··· | 21,959,043 (23.4%) | ··· |
| 35-44 | ··· | ··· | 19,096,807 (20.3%) | ··· |
| 45-54 | ··· | ··· | 17,037,165 (18.1%) | ··· |
| 55-64 | ··· | ··· | 15,700,713 (16.7%) | ··· |
| 65-74 | ··· | ··· | ··· | 12,569,714 (47.0%) |
| 75-84 | ··· | ··· | ··· | 8,825,326 (33.0%) |
| ≥85 | ··· | ··· | ··· | 5,323,071 (19.9%) |
| **Race and ethnicity** |  | | | |
| Hispanic, any race | 23,919,528 (15.7%) | 8,334,426 (26.3%) | 13,228,368 (14.1%) | 2,356,733 (8.8%) |
| Non-Hispanic, Black | 35,632,730 (23.4%) | 8,374,515 (26.4%) | 24,175,496 (25.7%) | 3,082,719 (11.5%) |
| Non-Hispanic Other | 4,472,256 (2.9%) | 1,200,030 (3.8%) | 2,294,975 (2.4%) | 977,250 (3.7%) |
| Non-Hispanic, White | 88,424,926 (58.0%) | 13,805,754 (43.5%) | 54,317,762 (57.8%) | 20,301,409 (76.0%) |
| **Geographic census region** |  | | | |
| Northeast | 21,295,859 (14.0%) | 4,217,838 (13.3%) | 12,695,069 (13.5%) | 4,382,952 (16.4%) |
| Midwest | 33,528,031 (22.0%) | 7,079,006 (22.3%) | 20,382,776 (21.7%) | 6,066,249 (22.7%) |
| South | 67,065,134 (44.0%) | 14,693,094 (46.3%) | 41,778,892 (44.4%) | 10,593,148 (39.6%) |
| West | 30,560,415 (20.0%) | 5,724,787 (18.1%) | 19,159,865 (20.5%) | 5,675,762 (21.2%) |
| **Residence in metropolitan statistical area** | 127,965,999 (83.9%) | 26,991,719 (85.1%) | 79,395,431 (84.4%) | 21,578,848 (80.8%) |

**Supplemental Table 2.** Most frequent indications among visits with inappropriate antibiotic prescribing and a plausible antibiotic indication.

| **Indication** | **Overall, No. (%)** (n = 22,736,645) | **Children, No. (%)**  (n = 5,289,605) | **Adults, No. (%)**  (n = 14,078,131) | **Older adults, No. (%)**  (n = 3,368,909) |
| --- | --- | --- | --- | --- |
| Acute serous or nonsuppurative otitis media | 674,203 (3.0%) | 557,313 (10.5%) | 113,357 (0.8%) | 3,532 (0.1%) |
| Asthma | 1,316,811 (5.8%) | 168,216 (3.2%) | 1,061,136 (7.5%) | 87,458 (2.6%) |
| Bronchitis | 3,967,168 (17.4%) | 637,318 (12.0%) | 2,742,513 (19.5%) | 587,336 (17.4%) |
| Conjunctivitis, scleritis, and other ophthalmologic conditions | 691,978 (3.0%) | 360,901 (6.8%) | 279,095 (2.0%) | 51,981 (1.5%) |
| Fungal infection | 183,021 (0.8%) | 56,194 (1.1%) | 108,047 (0.8%) | 18,779 (0.6%) |
| Other viral infection | 1,112,529 (4.9%) | 290,852 (5.5%) | 641,537 (4.6%) | 180,138 (5.3%) |
| Parasitic infection | 72,720 (0.3%) | 47,207 (0.9%) | 25,512 (0.2%) | 0 (0.0%) |
| Potential signs and symptoms of infection | 12,287,509 (54.0%) | 2,388,468 (45.2%) | 7,703,768 (54.7%) | 2,195,272 (65.2%) |
| Skin and soft tissue infection | 73,196 (0.3%) | 15,982 (0.3%) | 40,677 (0.3%) | 16,536 (0.5%) |
| Toxic gastroenteritis and colitis | 0 (0.0%) | 0 (0.0%) | 0 (0.0%) | 0 (0.0%) |
| Upper respiratory infection | 2,357,505 (10.4%) | 767,149 (14.5%) | 1,362,484 (9.7%) | 227,872 (6.8%) |

**Supplemental Table 3.** Most frequent diagnosis codes among visits with inappropriate antibiotic prescribing in which the indication was potential signs and symptoms of infection.

| **Overall** | **Children** | **Adults** | **Older adults** |
| --- | --- | --- | --- |
| 1. R109: Unspecified abdominal pain  2. R51: Headache  3. R112: Nausea with vomiting, unspecified  4. R060: Dyspnea  5. R509: Fever, unspecified  6. R111: Vomiting  7. R197: Diarrhea, unspecified  8. R101: Pain localized to upper abdomen  9. R05: Cough  10. R103: Pain localized to other parts of lower abdomen | 1. R509: Fever, unspecified  2. R111: Vomiting  3. R109: Unspecified abdominal pain  4. R05: Cough  5. R197: Diarrhea, unspecified  6. R112: Nausea with vomiting, unspecified  7. R51: Headache  8. R21: Rash and other nonspecific skin eruption  9. R103: Pain localized to other parts of lower abdomen  10. R098: Other specified symptoms and signs involving the circulatory and respiratory systems | 1. R109: Unspecified abdominal pain  2. R51: Headache  3. R112: Nausea with vomiting, unspecified  4. R101: Pain localized to upper abdomen  5. R060: Dyspnea  6. R103: Pain localized to other parts of lower abdomen  7. R197: Diarrhea, unspecified  8. R111: Vomiting  9. R110: Nausea  10. R05: Cough | 1. R060: Dyspnea  2. R109: Unspecified abdominal pain  3. R197: Diarrhea, unspecified  4. R112: Nausea with vomiting, unspecified  5. R51: Headache  6. R101: Pain localized to upper abdomen  7. R05: Cough  8. R509: Fever, unspecified  9. R111: Vomiting  10. R110: Nausea |

**Supplemental Table 4.** Most frequent diagnosis codes among visits with inappropriate antibiotic prescribing and no plausible antibiotic indication.

| **Overall** | **Children** | **Adults** | **Older adults** |
| --- | --- | --- | --- |
| 1. I10: Essential (primary) hypertension  2. R079: Chest pain, unspecified  3. M255: Pain in joint  4. R078: Other chest pain  5. M796: Pain in limb, hand, foot, fingers and toes  6. F419: Anxiety disorder, unspecified  7. R55: Syncope and collapse  8. M545: Low back pain  9. F329: Major depressive disorder, single episode, unspecified  10. R42: Dizziness and giddiness | 1. S099: Unspecified injury  of face and head  2. M255: Pain in joint  3. K590: Constipation  4. S934: Sprain of ankle  5. R458: Other symptoms and signs involving emotional state  6. K529: Noninfective gastroenteritis and colitis, unspecified  7. S060: Concussion  8. F329: Major depressive disorder, single episode, unspecified  9. M796: Pain in limb, hand, foot, fingers and toes  10. T148: Other injury of unspecified body region | 1. R079: Chest pain, unspecified  2. I10: Essential (primary) hypertension  3. M255: Pain in joint  4. R078: Other chest pain  5. F419: Anxiety disorder, unspecified  6. M796: Pain in limb, hand, foot, fingers and toes  7. M545: Low back pain  8. F329: Major depressive disorder, single episode, unspecified  9. R458: Other symptoms and signs involving emotional state  10. F179: Nicotine dependence | 1. I10: Essential (primary) hypertension  2. R079: Chest pain, unspecified  3. M255: Pain in joint  4. R55: Syncope and collapse  5. I489: Unspecified atrial fibrillation and atrial flutter  6. R42: Dizziness and giddiness  7. E119: Type 2 diabetes mellitus without complications  8. R531: Weakness  9. I509: Heart failure, unspecified  10. N179: Acute kidney failure, unspecified |

**Supplemental Table 5.** Most frequent indications among visits with inappropriate antibiotic prescribing and a plausible antibiotic indication, main versus sensitivity analysis.

| **Subcategory** | **Overall, Main Analysis, No. (%)** (n = 22,736,645) | **Overall, Sensitivity Analysis, No. (%)** (n = 22,736,645) |
| --- | --- | --- |
| Acute serous or nonsuppurative otitis media | 674,203 (3.0%) | 625,618 (2.8%) |
| Asthma | 1,316,811 (5.8%) | 1,439,330 (6.3%) |
| Bronchitis | 3,967,168 (17.4%) | 3,778,622 (16.6%) |
| Conjunctivitis, scleritis, and other ophthalmologic conditions | 691,978 (3.0%) | 695,108 (3.1%) |
| Fungal infection | 183,021 (0.8%) | 167,607 (0.7%) |
| Other viral infection | 1,112,529 (4.9%) | 1,090,870 (4.8%) |
| Parasitic infection | 72,720 (0.3%) | 73,958 (0.3%) |
| Potential signs and symptoms of infection | 12,287,509 (54.0%) | 12,410,736 (54.6%) |
| Skin and soft tissue infection | 73,196 (0.3%) | 75,501 (0.3%) |
| Toxic gastroenteritis and colitis | 0 (0.0%) | 0 (0.0%) |
| Upper respiratory infection | 2,357,505 (10.4%) | 2,379,291 (10.5%) |

**Supplemental Figure 1.** Number of weighted visits with antibiotic prescriptions and inappropriate prescribing with or without a plausible antibiotic indication.


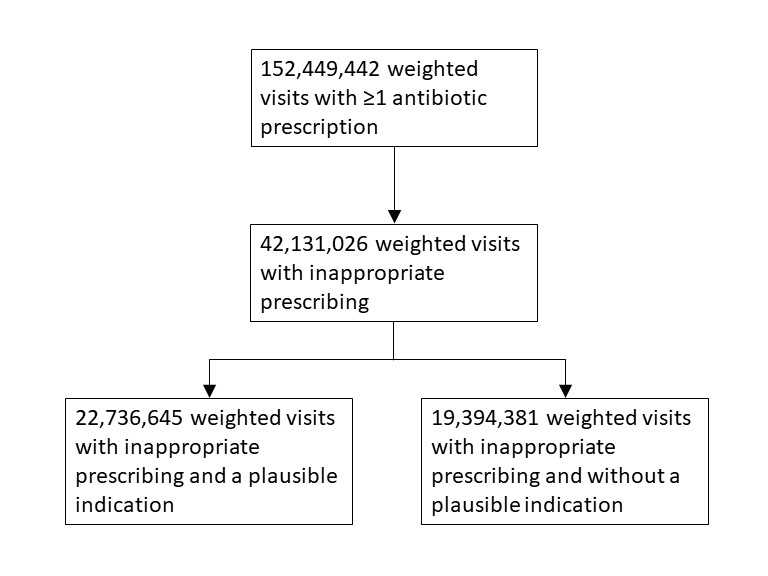

Supplement: Ladines-Lim et al. supplementary material 1 — Ladines-Lim et al. supplementary material [file S2732494X24000792sup001.docx]
