# Supplementary figures and images for: Appropriateness of Antibiotic Prescribing in US Emergency Department Visits, 2016–2021
Source: Antimicrob Steward Healthc Epidemiol. 2024 May 14;4(1):e79. doi: 10.1017/ash.2024.79 (PMC11094377; doi:10.1017/ash.2024.79)

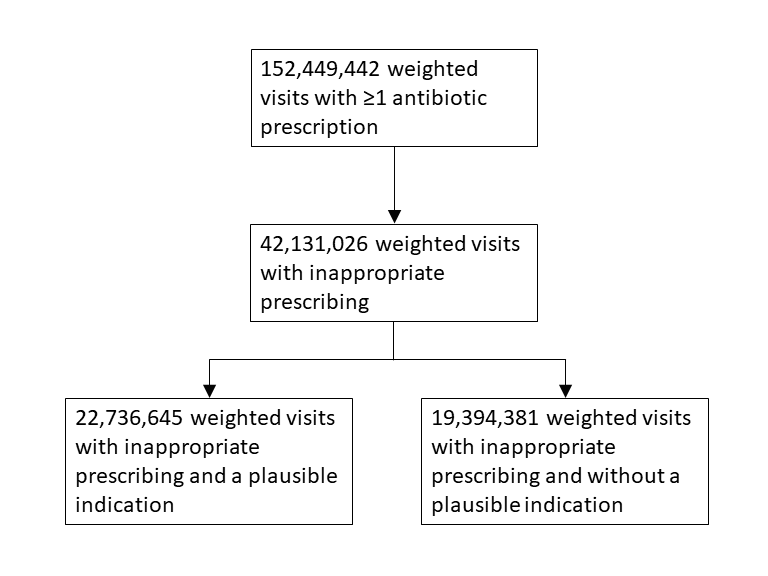

Supplement: Ladines-Lim et al. supplementary material 2 — Ladines-Lim et al. supplementary material [file S2732494X24000792sup002.tif]
